# Association Between Muscle Activity of Upper Limbs and Respiratory Parameters During Functional Performance in People With Chronic Obstructive Pulmonary Disease

**DOI:** 10.1155/oti/3023322

**Published:** 2025-03-25

**Authors:** Ching-Yun Chen, Chieh-Hsiang Hsu, Sheng-Han Tsai, Cheng-Feng Lin, Yu-Chen Lin, Hsiu-Yun Hsu, Chiung-Zuei Chen, Li-Chieh Kuo

**Affiliations:** ^1^Institute of Allied Health Sciences, College of Medicine, National Cheng Kung University, Tainan, Taiwan; ^2^Department of Mechanical Engineering, Southern Taiwan University of Science and Technology, Tainan, Taiwan; ^3^Department of Internal Medicine, National Cheng Kung University Hospital, Tainan, Taiwan; ^4^Department of Physical Therapy, College of Medicine, National Cheng Kung University, Tainan, Taiwan; ^5^Department of Occupational Therapy, Da-Yeh University, Dacun, Changhua County, Taiwan; ^6^Department of Physical Medicine and Rehabilitation, National Cheng Kung University Hospital, Tainan, Taiwan; ^7^Department of Occupational Therapy, College of Medicine, National Cheng Kung University, Tainan, Taiwan; ^8^Medical Device Innovation Center, National Cheng Kung University, Tainan, Taiwan

**Keywords:** accessory inspiratory muscles, activities of daily living (ADLs), chronic obstructive pulmonary disease (COPD), respiratory expenditure

## Abstract

**Background:** Few studies have examined the activity of the accessory inspiratory muscles as well as respiratory function simultaneously in individuals with chronic obstructive pulmonary disease (COPD) while performing activities of daily living (ADLs). This cross-sectional study is aimed at understanding the differences in the demands for respiratory expenditure and activity of the upper limbs and accessory inspiratory muscles during functional performance in individuals with and without COPD.

**Methods:** Thirteen patients with mild to moderate COPD and 10 healthy adults were enrolled. All participants were asked to complete the requested ADL tasks involving upper limb elevation while recording activities of muscles of the accessory inspiratory muscles, as well as respiratory expenditure via the Delsys Trigno electromyography and Ultima CardiO2 system, respectively.

**Results:** Muscle activity of the pectoralis major (PM) in the COPD group was significantly higher than that in the non-COPD group during washing both sides of the head (*p* < 0.05) and storing 2- and 4-kg objects on a shoulder-height shelf (*p* < 0.05). Ventilatory inefficiency and metabolic expenditure were significantly higher during the storage of objects at head height in the COPD group. A positive correlation was observed between sternocleidomastoid muscle activity and metabolic/ventilatory expenditure in the non-COPD group. However, muscle activity of the upper trapezius was significantly correlated with metabolic/ventilatory expenditure in the COPD group. Higher PM muscle activity and ventilatory inefficiency in the COPD group were found during performing ADLs involving upper limb elevation.

**Conclusions:** Individuals with COPD demonstrated increased accessory inspiratory muscle activity, reduced ventilatory efficiency, and higher metabolic expenditure during ADLs involving upper limb elevation compared to healthy controls. The PM in the COPD group was the major accessory inspiratory muscle for performing ADLs involving upper limb elevation. These findings could inform recommendations for individuals with COPD to adjust their strategies for upper limb elevation while performing ADLs. Training of the larger accessory inspiratory muscles in rehabilitation programs has also been considered to enhance ADL performance in individuals with COPD.

**Trial Registration:** ClinicalTrials.gov identifier: NCT04146948

**Summary**

• **Current knowledge:** Most of our ADLs involve upper limb elevation, which restricts the expansion of the rib cage and consequently reduces the efficiency of the ventilatory response. People with COPD thus experience dyspnea more easily when performing ADLs involving upper limb elevation than when performing functional tasks using only the lower limbs. However, only few studies have examined the activity of the upper limbs and accessory inspiratory muscles as well as respiratory function simultaneously in individuals with COPD during their daily tasks.
• **What this paper contributes to our knowledge:** The activation of the accessory inspiratory muscles, especially the pectoralis major, in the COPD group was higher, and the ventilatory efficiency in the COPD group was worse than that in the non-COPD group during ADLs with upper limb involvement. The findings of this study represented people with COPD would use compensatory strategies to carry out ADLs. This study also suggests clinical practitioners should integrate suitable accessory inspiratory muscle training into rehabilitation programs for patients with COPD to enhance their ADL performance and life quality.

## 1. Introduction

Chronic obstructive pulmonary disease (COPD) is a preventable and treatable chronic inflammatory lung disease that causes airflow restriction in obstructed airways, commonly due to chronic bronchitis and/or emphysema [1]. With medical advancements, extended life expectancy has increased the prevalence of COPD and, subsequently, the economic burden of COPD care. COPD affects not only lung function but also functional capacity, which could be related to independence in activities of daily living (ADLs) that might interfere with quality of life [2].

Disruption of independence in ADLs is a multifactorial issue for people with COPD. Perceiving dyspnea is the most prominent problem among individuals in this group and might be associated with increases in metabolic and ventilatory demands while performing ADLs [3]. This refers to the subjective sensation of difficult or uncomfortable breathing, which affects functional performance in people with COPD. A previous study reported that oxygen uptake increased by approximately 55% of the maximal oxygen uptake and ventilation increased by 60% of maximal voluntary ventilation during the performance of ADLs in COPD [4]. Respiratory constraints make patients with COPD unable to properly respond to the metabolic and ventilatory demands of ADLs. More than half of them had difficulty completing ADLs independently and needed assistance to accomplish some of the tasks [5, 6].

Muscle dysfunction is another physiological problem associated with COPD. Alterations in muscle properties, including muscle size and fiber type, have been reported to be affected by COPD progression [7]. Thus, muscular strength and endurance limit the physical activity of individuals with COPD. A previous report indicated that their muscle strength was generally lower than that of age-matched healthy adults [5]. Decreased muscle function contributes to an inactive lifestyle, which might result in a vicious cycle in people with COPD.

Most of our ADLs involve upper limb elevation, which restricts the expansion of the rib cage [8] and consequently reduces the efficiency of the ventilatory response. Therefore, individuals with COPD experience dyspnea more easily when performing ADLs involving upper limb elevation than when performing functional tasks using only the lower limbs [9]. For this reason, individuals with COPD may activate their accessory inspiratory muscles as a compensatory strategy to respond to excessive ventilatory demands while performing functional tasks using their upper limbs. The accessory inspiratory muscles, including the scalene, sternocleidomastoid (SCM), upper trapezius (UT), serratus anterior, pectoralis major (PM) and minor, latissimus dorsi (LD), and erector spinae [10], participate in stabilizing the shoulder girdle and assist in breathing during upper limb elevation [9]. Consequently, the accessory inspiratory muscles play an important role in both ventilation and ADLs involving upper limb elevation in patients with COPD.

Metabolic and ventilatory parameters have been investigated to determine respiratory limitations during ADL performance in patients with COPD [3–5, 11]. Most previous studies have analyzed respiratory parameters and muscle function separately. Few studies have simultaneously examined muscle activity and respiratory function during the performance of functional tasks. Some studies have examined muscle activation while performing tasks related to arm movements but not ADLs [12]. As COPD can potentially alter muscle status and function, it was difficult to identify whether the difference in muscle activation between people with and without COPD affected ADL performance.

This study is aimed at investigating the differences in muscle activity, including accessory inspiratory and respiratory parameters during the performance of ADLs. Participants with mild to moderate COPD were the target group in the study because they could still complete most ADLs but with a certain degree of difficulty. This study also examined the association between factors related to an increase in accessory inspiratory muscle activity and metabolic and ventilatory expenditures in people with and without COPD during ADLs with upper limb elevation. This study hypothesized that the COPD group would exhibit higher respiratory parameters and accessory inspiratory muscle activity compared to the non-COPD elderly group across different ADL tasks. Additionally, it was expected that accessory inspiratory muscle activity in both groups would be associated with respiratory parameters, including metabolic and ventilatory factors.

## 2. Methods

### 2.1. Study Design

This was a cross-sectional and observational study comparing the muscle activity of the accessory inspiratory muscles and respiratory expenditure between patients with COPD and age- and sex-matched healthy participants while performing simulated ADL tasks. This study was conducted and reported in accordance with the STROBE (Strengthening the Reporting of Observational Studies in Epidemiology) guidelines for cross-sectional studies.

### 2.2. Participants

Convenience and snowball sampling was used to recruit the participants with and without COPD. Thirteen participants with COPD were recruited from the Department of Internal Medicine at National Cheng Kung University Hospital. Non-COPD participants were recruited from the local community and matched for age and sex. The inclusion criteria for the participants with COPD were as follows: (1) 40 years or older with a clinical diagnosis of COPD and severity classification according to the Global Initiative for Chronic Obstructive Lung Disease (GOLD) guidelines from Stages I to IV and (2) stable medical state (no acute deterioration due to exacerbation of COPD or other medical problems within 1 month before the evaluation). The exclusion criteria for participants with COPD were as follows: (1) clinical diagnosis of cancer or asthma; (2) cardiovascular, musculoskeletal, or neurological diseases limiting physical activities in daily life; and (3) inability to follow instructions during the experiment. The inclusion and exclusion criteria for participants without COPD were the same as those for participants with COPD, except for the clinical diagnosis of COPD. All recruited participants were informed of the purpose and experimental details of the study and signed consent forms. This study was approved by the Institutional Review Board of National Cheng Kung University Hospital. The sample size for this study was determined based on previous research by Baarends et al. [13], which examined metabolic and ventilatory responses in patients with COPD and healthy age-matched subjects. Additionally, practical constraints, including the time available for participant recruitment and data collection, influenced the final number of participants.

### 2.3. Simulated ADL Tasks

**Figure 1:**
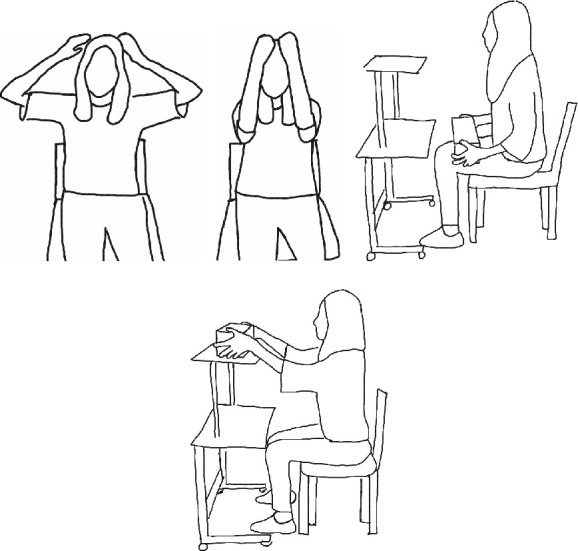
Two simulated tasks were performed in this study: (a) washing the top of the head, (b) washing both sides of the head, (c) the starting position of the storing tasks, and (d) lifting the objects up to the shelf.

Based on previous studies, two ADL tasks that have higher performance challenges and commonly cause dyspnea in people with COPD were chosen as the simulated ADL conditions in this study [3, 14–16]. These two simulated tasks included washing hair and storing objects on a shelf (Figure 1). The hair washing task was divided into two steps: washing the top and both sides of the head (Figures 1(a) and 1(b)). The storage tasks consisted of three 2-kg objects and three 4-kg objects up to the shoulder and head height of the shelf. This task was divided into two steps: storing the objects on the shelf and lowering both upper limbs to the thigh (Figures 1(c) and 1(d)). Only the step of storing objects on the shelf was chosen for analysis. The object size was 31 × 22.8 × 10.3 cm, that is, a regular-sized carton, requiring participants to use both hands for the storage task. All tasks were performed in the sitting position to diminish the influence of lower limb movement. To eliminate movement variability among the participants, every examinee was requested to follow video instructions for each task. Each simulated task was repeated thrice. The rest interval between performing the simulated tasks depended on the real-time display of pulse oximetry saturation (SpO_2_) and the perception of dyspnea. All muscle activities and respiratory expenditures of each participant were recorded simultaneously while performing the simulated ADL tasks.

### 2.4. Measurements of Respiratory Expenditures

Respiratory expenditures, including oxygen consumption, carbon dioxide production, and ventilatory demand, were assessed using the CPX Ultima CardiO2 gas exchange analysis system (Medical Graphics Corp., St. Paul, Minnesota, United States). Forced expiratory volume in 1 s (FEV_1_), forced vital capacity (FVC), minute ventilation (*V*_E_), oxygen consumption (*V*_o2_), carbon dioxide production (*V*_co2_), maximal oxygen consumption (*V*_o2⁣max_), and maximum voluntary ventilation (MVV) were measured during the test. FEV_1_ and FVC indicate the subject's pulmonary status. VE indicates the volume of air breathed per minute. *V*_o2_ and *V*_co2_ indicate the amount of metabolic expenses. *V*_o2⁣max_ indicates the subject's maximal exercise capacity. MVV indicates the subject's maximal breathing capacity. VE/MVV and *V*_o2_/*V*_o2⁣max_ were also calculated in the study, representing the subject's ventilatory reserve and metabolic reserve, respectively. These respiratory parameters were calculated using the Breeze Suite 8.6 system (Medical Graphics Corp., St. Paul, Minnesota, United States). Participants were required to wear and breathe through a facial cover so that gas could be collected for measurement.

### 2.5. Assessments of Muscle Activities of the Accessory Inspiratory

Muscle activity, including the accessory inspiratory was recorded using a surface electromyography (sEMG) system (Trigno Mini Sensor, Delsys Inc., Natick, Massachusetts, United States). The minihead electrodes were attached and paralleled to the target muscles, with the references of electrodes attached over the spinous process or sternum. Before electrode attachment, the skin was cleaned with an alcohol wipe. The minihead and reference electrodes were fixed with tape to reduce artifacts. The sEMG sampling rate was 2000 Hz, and the raw data were band-pass filtered with a sixth Butterworth filter (20–500 Hz). Root mean square (RMS) amplitude was calculated with a window length of 100 ms. The accessory inspiratory muscles include SCM, UT, PM, and LD. The ratio of the sEMG data during simulated ADL tasks to sEMG data during maximum voluntary contraction (MVC) was used to compare muscle activity, expressed as the muscle activity ratio level (ARL). The EMG electrodes were positioned according to the guidelines outlined in Cram's Introduction to sEMG [17]. The RMS of MVC and each simulated ADL task was calculated. All participants performed MVC in a sitting position, with the MVC of each target muscle performed three times. Each MVC was sustained for 3 s, followed by a 1-min rest. Only the dominant side of the accessory inspiratory muscles was tested in this study.

### 2.6. Data and Statistical Analysis

All data were analyzed using SPSS software (Version 17.0; SPSS Inc., Chicago, Illinois, United States). All values of *p* < 0.05 were considered statistically significant. The characteristics of the COPD and age- and sex-matched groups were given as mean and standard deviation, including age, sex, body mass index, FEV_1_, FEV_1% predicted_, FVC, FVC_% predicted_, FEV_1_/FVC, MVV, and *V*_O2⁣max_. GOLD stages were presented according to the number of participants with COPD. Differences between the two groups in characteristics, sEMG signals, and respiratory parameters during each simulated ADL task were tested using the Mann–Whitney *U* test. Spearman's rank correlation coefficient was used to represent the relationship among the parameters of muscle activation and respiratory performances, with the analyses conducted separately for the COPD and non-COPD groups. Missing data was handled by pairwise deletion, reducing the sample size.

## 3. Results

### 3.1. Demographic Characteristics of Participants

Thirteen participants with COPD and 10 age- and sex-matched participants without COPD were recruited for the study. The COPD group consisted of eight subjects with GOLD I, three with GOLD II, and two with GOLD III. The demographic and clinical characteristics of both groups are presented in Table 1. There were no statistically significant differences between the two groups in terms of age, BMI, FEV_1_, FVC, FEV_1_/FVC, and MVV. However, FEV_1_/FEV_1 predicted_ and *V*_O2⁣max_ were significantly different between the groups (*p* < 0.05).

### 3.2. Respiratory and Muscle Function During Task Performance

Table 2 shows the ARL of the accessory inspiratory muscles and respiratory parameters during the hair washing task between the two groups. Only the ARL of the PM during Step 2 of the hair washing task showed a statistically significant difference between the two groups. *V*_E_/*V*_O2_ and *V*_E_/*V*_O2_ during Step 1 of the hair washing task were significantly different between the two groups.

The lower part of Table 2 also shows the ARL of the accessory inspiratory muscles and respiratory parameters during the storage task between the two groups. The ARL of the PM in the COPD group was significantly higher than that in the non-COPD group when storing 2- and 4-kg objects up to the shoulder height of the shelf (*p* < 0.05). *V*_E_/*V*_O2_ and *V*_E_/*V*_CO2_ during storing 2-kg objects up to shoulder height and head height of the shelf were significantly different between the two groups (*p* < 0.05). *V*_E_/*V*_CO2_ during storing 4-kg objects up to the shoulder height of the shelf was significantly different between the two groups (*p* < 0.05). *V*_O2_/*V*_O2⁣max_ was significantly different between the two groups when storing 4-kg objects up to the shoulder height of the shelf (*p* < 0.05).

### 3.3. Relationships Between Respiratory and Muscle Function During Task Performance

Table 3 shows the correlation between the ARL of the accessory inspiratory muscles and metabolic and ventilatory expenditure during hair washing and storage tasks. In the results of hair washing and storage tasks, the ARL of the SCM was significantly correlated with *V*_O2_/*V*_O2⁣max_ and *V*_E_/MVV in the non-COPD group. Only the ARL of the UT had a positive correlation with *V*_O2_/*V*_O2⁣max_ in the non-COPD group during storage of 4-kg objects to the shoulder height of the shelf. The results showed a correlation between the ARL of the UT and *V*_E_/MVV in the COPD group during storage tasks.

## 4. Discussion

The exercise capacity of the COPD group was lower than that of the non-COPD group in this study. *V*_O2⁣max_ indicates the maximal oxygen uptake during incremental exercise, which represents the exercise capacity of the participants. Demographic data showed that *V*_O2⁣max_ was significantly different between the two groups. FEV_1_/FEV_1 predicted_ (%) was used to stratify the severity of COPD in the clinical diagnosis. As this study recruited COPD patients with GOLD Stages I–III, FEV_1_/FEV_1 predicted_ (%) in the COPD group was significantly lower than that in the non-COPD group. In general, the ARL of the target muscles in the COPD group during the simulated ADLs was higher than that in the non-COPD group; however, some differences were not significant. The ARL of the PM in the COPD group during Step 2 of hair washing was significantly higher than that in the non-COPD group. The results were not similar to those of a previous study that requested participants to maintain shoulder flexion and move the forearm to execute instrumental ADLs, such as writing on broad and shaking hands [12]. The UT is the major muscle that maintains the shoulder girdle during these tasks. However, unlike in the previous study which assessed the writing or shaking hands' tasks, the participants in this study moved both upper limbs to perform washing of both sides of the head. The COPD group with poor ventilatory capacity used a compensatory approach to deal with tasks involving upper limb elevation. Therefore, when the PM is passively extended and the chest wall is in an expanded position [18] during the washing of both sides of the head, this position shortens the neck accessory muscles, which leads to a less effective response to ventilation. Therefore, the PM in the COPD group had to contract more to respond to the ventilation demand and overcome the decreased activity of the neck accessory muscles.

According to the descriptive statistics, the respiratory parameters in the COPD group were higher than the values in non-COPD group except to the washing hair task. This simulated ADL task might be the works with mild difficulty level for both groups. However, the respiratory parameters showed that ventilatory efficiency (*V*_E_/*V*_O2_ and *V*_E_/*V*_CO2_) was significantly worse in the COPD group than in the non-COPD group during Step 1 of the hair washing task. During Step 1 of the hair washing task, both upper limbs were positioned in front of the trunk, which would alter inspiratory capacity [8, 13]. This position reduced inspiratory capacity and lung volume in the COPD group [18]. However, the constraint of thoracic cage expansion with both upper limbs on the side was less than that with the upper limbs in front of the trunk. The thoracic cage was expanded, which increased the chest wall volume and made ventilation more efficient. Therefore, the ventilatory efficiency between the two groups did not show a significant difference during Step 2 of the hair washing task.

The ARL of the PM in the COPD group was significantly higher than that in the non-COPD group when storing 2- and 4-kg objects at shoulder height of the shelf but not at head height. A previous study pointed out that accessory inspiratory muscles would perform dual tasks, including ventilation and shoulder girdle support, during upper limb elevation [1]. To meet the ventilatory requirements of the tasks, the COPD group had to achieve more PM recruitment while storing objects at shoulder height on the shelf. With increased upper limb elevation, the non-COPD group used the SCM, which is smaller than the PM and UT in terms of muscle size, to achieve the required movements. However, the COPD group preferred to use the larger muscle group to deal with the task, but the activity in these muscles did not differ significantly between the two groups.

The ventilatory equivalent for O_2_ in the COPD group was significantly higher than that in the non-COPD group when storing 2 kg of objects at shoulder and head height. The ventilatory equivalent for CO_2_ in the COPD group was also significantly higher than that in the non-COPD group during storage tasks, except for storing 4-kg objects to the head-height shelf. The COPD group showed worse ventilatory efficiency than the non-COPD group during storage of the object on the shelf. Because the participants needed to raise both upper limbs up to the shelf in front of them during the storage tasks, this position prevented the thoracic cage from expanding. In other words, ventilatory efficiency was poor during storage tasks. However, the efficiency was not different between the two groups only when storing the heaviest and highest objects, which might be due to the workload causing a respiratory burden for both groups.

Progressive metabolic and ventilatory costs were not incurred when the weight and height of the ADL tasks increased. The metabolic demand in the COPD group was significantly higher than that in the non-COPD group when storing 4-kg objects on the shelf at head height. This is because storing heavier objects on a higher shelf resulted in a higher proportion of oxygen consumption in the COPD group than in the non-COPD group. Since previous studies have indicated that large muscles used during movement would lead to significant energy expenditure [4], the COPD group in this study used larger muscles (such as the PM and UT) to excute the higher upper limb elevation tasks and contribute to higher energy expenditure.

The prior research works indicated the arm elevation was associated with an increase in metabolic and ventilatory rate in both non-COPD and COPD populations [8, 13, 19]. The non-COPD and COPD groups needed to make more efforts on metabolism and ventilation during performing ADLs which was evidenced in *V*_O2_/*V*_O2⁣max_ and *V*_E_/MVV. The SCM in the non-COPD group and the UT in the COPD group might thus be used to respond to the requirement of ventilation while performing tasks with arm lifting. The non-COPD group primarily relied on relatively smaller accessory respiratory muscles, such as the SCM, to assist in performing activities. In contrast, the COPD group required the engagement of larger muscles, such as the UT, to support the execution of ADLs. The findings suggested that the SCM in the non-COPD group and the UT in the COPD group might play a compensatory role in responding to these ventilatory requirements.

### 4.1. Limitations

The lack of diversity in the COPD populations and the small sample size were the limitations of this study. Most patients enrolled had mild to moderate COPD; no participants with severe COPD were included. Although the trends were observed, such as higher values of accessory inspiratory muscle activity and respiratory parameters in the COPD group compared to the non-COPD group, most differences did not reach statistical significance. These findings highlighted the need for further investigation with larger sample sizes to better understand these observed trends and their potential clinical implications. The recruitment of only male subjects may not represent overall performance. Owing to the attachment of the sEMG sensors, the participants only pretended to wash their hair to reduce sEMG signal contamination by external factors. Performing the simulated ADLs may not reflect 100% muscle activation and respiratory demands of participants in reality. In addition, the participants were asked to follow the sequence of movements while performing tasks, but the movement would not be the same as that in real life. Finally, we analyzed only the mean muscle activity during the tasks. This would only present parts of the recruitment pattern of the accessory inspiratory muscles.

## 5. Conclusions

This study showed that muscle recruitment and respiratory expenses differed between the COPD and non-COPD groups. The activation of the accessory inspiratory muscles, especially the PM, in the COPD group was higher, and the ventilatory efficiency in the COPD group was worse than that in the non-COPD group during ADLs with upper limb involvement. Moreover, the different accessory inspiratory muscles were correlated with metabolic and ventilatory expenditures between the two groups but with different performance patterns. Thus, the findings of this study might explain why people with COPD would use a compensatory way to carry out ADLs and why clinical practitioners should integrate suitable accessory inspiratory muscle training into rehabilitation programs for patients with COPD, which may enhance their ADL performance and quality of life.

## Figures and Tables

**Table 1:** The demographic data and clinical characteristics in the COPD and non-COPD groups.

	**Non-COPD**		**COPD**		**p** ** value**
Number	10		13		—
Sex (% male)	100%		100%		—
Age (years, *M* SD)	68.1	(4.2)	63.8	(9.5)	0.148
BMI (kg∙m^2^)	24.3	(0.8)	24.0	(4.1)	0.410
FEV_1_ (L)	2.3	(0.6)	1.9	(0.7)	0.284
FEV_1_/FEV_1 predicted_ (%)	83.4	(19.2)	66.9	(19.3)	0.042⁣^∗^
FVC (L)	3.1	(0.6)	2.9	(0.8)	0.446
FVC/FVC _predicted_ (%)	92.6	(16.4)	92.6	(16.4)	0.057
FEV_1_/FVC (%)	67.5	(12.2)	79.7	(15.5)	0.927
MVV (L/min)	98.0	(29.2)	81.6	(40.0)	0.410
*V*_O2⁣max_ (mL·min^−1^·kg^−1^)	22.4	(4.8)	17.4	(5.1)	0.042⁣^∗^
GOLD I/II/III/IV (*n*)	—		8/3/2/0		—

Abbreviations: FEV_1_, forced expiratory volume in 1 s; FEV_1 predicted_, predicted FEV_1_ value; FVC, forced vital capacity; FVC _predicted_, predicted FVC value; GOLD, Global Initiative for Chronic Obstructive Lung Disease; MVV, maximum voluntary ventilation; *V*_O2⁣max_, maximum rate of oxygen consumption.

⁣^∗^*p* ≤ 0.05, significant differences between the non-COPD and COPD groups.

**Table 2:** The activity ratio level (ARL) of accessory inspiratory muscles and respiratory parameters in the COPD and non-COPD groups during hair washing task and storing tasks.

	**Non-COPD**		**COPD**		**p** ** value**
Hair washing					
*Muscle activity*					
Step 1 (washing top of the head)					
SCM	2.91^b^	(0.96)	3.61	(2.51)	0.879
PM	7.39	(5.15)	10.21	(6.00)	0.166
UT	28.68	(6.87)	31.80	(9.94)	0.483
LD	8.31^a^	(5.46)	8.63^a^	(6.22)	1.000
Step 2 (washing both sides of the head)					
SCM	2.74	(1.63)	3.44	(2.76)	0.697
PM	3.18	(1.73)	7.47	(5.38)	0.042⁣^∗^
UT	29.37	(6.82)	33.29	(9.42)	0.284
LD	8.91^a^	(5.83)	9.03^a^	(6.71)	1.000
*Respiratory parameters*					
Step 1 (washing top of the head)					
*V*_O2_ (mL/kg/min)	4.64	(0.52)	4.55	(1.02)	0.738
*V*_CO2_ (mL/kg/min)	4.46	(0.62)	4.43	(1.17)	0.693
*V*_E_ (L/min)	10.88	(1.92)	13.00	(2.53)	0.057
*V*_O2_/*V*_O2⁣max_ (%)	21.76	(6.02)	28.41	(10.17)	0.067
*V*_E_/MVV (%)	14.01	(7.79)	20.25	(10.36)	0.115
*V*_E_/*V*_O2_	35.69	(5.86)	43.30	(7.07)	0.006⁣^∗^
*V*_E_/*V*_CO2_	37.28	(6.44)	44.71	(6.72)	0.008⁣^∗^
Step 2 (washing both sides of the head)					
*V*_O2_ (mL/kg/min)	4.75	(0.73)	4.39	(0.98)	0.208
*V*_CO2_ (mL/kg/min)	4.79	(0.98)	4.35	(1.25)	0.186
*V*_E_ (L/min)	12.86	(3.36)	13.05	(2.53)	0.924
*V*_O2_/*V*_O2⁣max_ (%)	22.14	(5.64)	27.34	(9.32)	0.738
*V*_E_/MVV (%)	15.30	(9.28)	20.24	(10.24)	0.313
*V*_E_/*V*_O2_	40.64	(5.40)	44.91	(7.55)	0.101
*V*_E_/*V*_CO2_	40.68	(5.34)	45.84	(7.66)	0.088
Storing tasks					
*Muscle activity*					
2-kg–shoulder height					
SCM	3.73	(2.24)	3.73	(2.13)	1.000
PM	19.34	(5.61)	31.35	(13.74)	0.010⁣^∗^
UT	33.08	(10.96)	39.16	(16.68)	0.313
LD	11.40	(5.64)	11.28^a^	(4.81)	1.000
2-kg–head height					
SCM	6.25	(4.17)	5.87	(3.15)	1.000
PM	21.80	(9.11)	29.69	(12.77)	0.166
UT	46.75	(7.29)	51.33	(16.81)	0.522
LD	11.91^a^	(6.12)	14.02^a^	(8.14)	0.651
4-kg–shoulder height					
SCM	5.14	(3.11)	5.32	(2.84)	0.927
PM	24.96	(7.91)	36.90	(14.44)	0.042⁣^∗^
UT	39.81	(10.82)	46.78	(20.10)	0.483
LD	12.78	(6.05)	12.47^a^	(5.26)	0.974
4-kg–head height					
SCM	8.07	(4.64)	7.33	(3.55)	0.976
PM	26.99	(8.96)	33.31	(11.74)	0.313
UT	54.21	(6.86)	55.68	(17.56)	0.832
LD	13.15^a^	(6.72)	15.51^a^	(9.11)	0.651
*Respiratory parameters*					
2-kg–shoulder height					
*V*_O2_ (mL/kg/min)	4.32	(0.44)	4.63	(1.11)	0.605
*V*_CO2_ (mL/kg/min)	4.19	(0.66)	4.29	(1.17)	1.000
*V*_E_ (L/min)	10.54	(1.82)	12.43	(2.50)	0.067
*V*_O2_/*V*_O2⁣max_ (%)	20.33	(5.63)	29.69	(14.60)	0.057
*V*_E_/MVV (%)	12.07	(5.17)	19.86	(11.35)	0.067
*V*_E_/*V*_O2_	36.59	(2.51)	40.54	(5.69)	0.042⁣^∗^
*V*_E_/*V*_CO2_	37.99	(3.10)	44.05	(6.38)	0.005⁣^∗^
2-kg–head height					
*V*_O2_ (mL/kg/min)	4.49	(0.91)	4.69	(1.27)	0.738
*V*_CO2_ (mL/kg/min)	4.07	(0.83)	4.31	(1.27)	0.738
*V*_E_ (L/min)	10.33	(2.53)	12.28	(2.07)	0.101
*V*_O2_/*V*_O2⁣max_ (%)	21.26	(7.93)	29.95	(15.09)	0.077
*V*_E_/MVV (%)	11.67	(4.88)	19.71	(11.77)	0.057
*V*_E_/*V*_O2_	34.55	(2.41)	39.67	(5.59)	0.012⁣^∗^
*V*_E_/*V*_CO2_	38.17	(3.17)	43.39	(6.14)	0.018⁣^∗^
4-kg–shoulder height					
*V*_O2_ (mL/kg/min)	4.53	(0.53)	4.66	(1.14)	1.000
*V*_CO2_ (mL/kg/min)	4.29	(0.63)	4.30	(1.22)	0.693
*V*_E_ (L/min)	10.87	(2.19)	12.37	(2.55)	0.131
*V*_O2_/*V*_O2⁣max_ (%)	21.23	(5.93)	29.23	(11.26)	0.067
*V*_E_/MVV (%)	12.33	(5.15)	19.32	(10.07)	0.115
*V*_E_/*V*_O2_	36.01	(3.51)	40.11	(6.27)	0.101
*V*_E_/*V*_CO2_	38.09	(2.87)	43.70	(6.68)	0.030⁣^∗^
4-kg–head height					
*V*_O2_ (mL/kg/min)	4.67	(0.79)	5.06	(1.32)	0.522
*V*_CO2_ (mL/kg/min)	4.16	(0.75)	4.61	(1.37)	0.563
*V*_E_ (L/min)	10.44	(2.39)	12.75	(2.63)	0.057
*V*_O2_/*V*_O2⁣max_ (%)	21.98	(7.23)	31.40	(10.99)	0.030⁣^∗^
*V*_E_/MVV (%)	11.92	(5.24)	20.00	(10.46)	0.067
*V*_E_/*V*_O2_	33.56	(2.68)	38.24	(6.59)	0.067
*V*_E_/*V*_CO2_	37.70	(2.97)	42.28	(6.82)	0.077

*Note:* Unit: %.

Abbreviations: AD, anterior deltoid; LD, latissimus dorsi; MD, middle deltoid; PM, pectoralis major; SCM, sternocleidomastoid; UT, upper trapezius.

^a^Missing value for one participant.

^b^Missing value for two participants.

⁣^∗^*p* ≤ 0.05, significant differences between the non-COPD and COPD groups.

**Table 3:** The correlation between metabolic and ventilatory expenditure and the ARL of accessory inspiratory muscles during the washing hair tasks and storing tasks in the non-COPD and COPD groups.

	**Non-COPD**				**COPD**			
	**V**_**O**2_/**V**_**O**2⁣max_	**p**	**V**_**E**_/**MVV**	**p**	**V**_**O**2_/**V**_**O**2⁣max_	**p**	**V**_**E**_/**MVV**	**p**
*Washing hair tasks*								
Step 1								
SCM	0.632^b^⁣^∗^	0.050	0.827^b^⁣^∗^	0.003	0.181	0.553	−0.231	0.448
PM	0.608	0.062	0.280	0.434	−0.121	0.694	0.071	0.817
UT	0.012	0.973	−0.328	0.394	0.258	0.354	0.500	0.082
LD	0.293^a^	0.444	−0.092^a^	0.729	−0.112^a^	0.814	−0.371^a^	0.236
Step 2								
SCM	0.681⁣^∗^	0.030	0.778⁣^∗^	0.008	−0.154	0.616	−0.346	0.247
PM	0.426	0.220	0.158	0.663	−0.192	0.529	−0.286	0.344
UT	−0.389	0.266	−0.377	0.283	0.324	0.280	0.538	0.058
LD	0.209^a^	0.589	0.025^a^	0.949	−0.189^a^	0.557	−0.427^a^	0.167
*Storing tasks*								
2-kg–shoulder height								
SCM	0.709⁣^∗^	0.022	0.842⁣^∗^	0.002	−0.176	0.566	−0.132	0.668
PM	0.430	0.214	0.224	0.533	−0.137	0.655	0.192	0.529
UT	0.552	0.098	0.297	0.405	0.170	0.578	0.566⁣^∗^	0.044
LD	0.588	0.074	0.297	0.405	−0.028^a^	0.931	−0.070^a^	0.829
2-kg–head height								
SCM	0.552	0.098	0.679⁣^∗^	0.025	−0.154	0.616	−0.269	0.374
PM	0.333	0.347	0.297	0.405	−0.110	0.721	−0.060	0.845
UT	−0.115	0.751	−0.406	0.244	0.412	0.162	0.495	0.086
LD	0.150^a^	0.700	0.083^a^	0.831	−0.028^a^	0.931	−0.196^a^	0.542
4-kg–shoulder height								
SCM	0.794⁣^∗^	0.006	0.818⁣^∗^	0.004	0.066	0.831	−0.198	0.517
PM	0.309	0.385	−0.006	0.987	0.038	0.901	0.236	0.437
UT	0.782⁣^∗^	0.008	0.455	0.187	0.099	0.748	0.385	0.194
LD	0.600	0.067	0.273	0.446	−0.056^a^	0.863	−0.259^a^	0.417
4-kg–head height								
SCM	0.491	0.150	0.479	0.162	0.077	0.803	−0.192	0.529
PM	0.006	0.987	0.091	0.803	−0.280	0.354	−0.137	0.655
UT	0.067	0.855	−0.370	0.293	0.527	0.064	0.577⁣^∗^	0.039
LD	0.250^a^	0.516	0.217^a^	0.576	−0.014^a^	0.966	−0.189^a^	0.557

Abbreviations: LD, latissimus dorsi; MVV, maximum voluntary ventilation; PM, pectoralis major; SCM, sternocleidomastoid; UT, upper trapezius; *V*_E_, minute ventilation; *V*_O2_, oxygen uptake; *V*_O2⁣max_, maximum rate of oxygen uptake.

^a^Missing value for one participant.

^b^Missing value for two participants.

⁣^∗^*p* ≤ 0.05, significant differences between the non-COPD and COPD groups.

## Data Availability

The datasets analyzed in this study are not publicly available due to data protection requirements. However, anonymized data or files may be available from the corresponding authors upon reasonable request.

## Supporting Information

Supporting Information Additional supporting information can be found online in the Supporting Information section. STROBE checklist for cross-sectional studies and the funding statement for production, COI statement for production, and data availability statement for production are included.

## References

[1] Celli B. R., MacNee W., Agusti A. (2004). Standards for the diagnosis and treatment of patients with COPD: a summary of the ATS/ERS position paper. *European Respiratory Journal*.

[2] Rabe K. F., Hurd S., Anzueto A. (2007). Global strategy for the diagnosis, management, and prevention of chronic obstructive pulmonary disease: GOLD executive summary. *American Journal of Respiratory and Critical Care Medicine*.

[3] Lahaije A. J., van Helvoort H., Dekhuijzen P. N. R., Heijdra Y. F. (2010). Physiologic limitations during daily life activities in COPD patients. *Respiratory Medicine*.

[4] Velloso M., Stella S. G., Cendon S., Silva A. C., Jardim J. R. (2003). Metabolic and ventilatory parameters of four activities of daily living accomplished with arms in COPD patients. *Chest*.

[5] Gosselink R., Troosters T., Decramer M. (2000). Distribution of muscle weakness in patients with stable chronic obstructive pulmonary disease. *Journal of Cardiopulmonary Rehabilitation*.

[6] Garrod R., Bestall J. C., Paul E. A., Wedzicha J. A., Jones P. W. (2000). Development and validation of a standardized measure of activity of daily living in patients with severe COPD: the London Chest Activity of Daily Living scale (LCADL). *Respiratory Medicine*.

[7] Miranda E. F., Malaguti C., Marchetti P. H., Dal Corso S. (2014). Upper and lower limb muscles in patients with COPD: similarities in muscle efficiency but differences in fatigue resistance. *Respiratory Care*.

[8] McKeough Z. J., Alison J. A., Bye P. T. P. (2003). Arm positioning alters lung volumes in subjects with COPD and healthy subjects. *The Australian Journal of Physiotherapy*.

[9] Celli B. R., Rassulo J., Make B. J. (1986). Dyssynchronous breathing during arm but not leg exercise in patients with chronic airflow obstruction. *New England Journal of Medicine*.

[10] Kendall F. P., McCreary E. K., Provance P. G. (2005). *Muscles: Testing and Function With Posture and Pain*.

[11] van Helvoort H. A., Willems L. M., Dekhuijzen P. N. R., van Hees H. W. H., Heijdra Y. F. (2016). Respiratory constraints during activities in daily life and the impact on health status in patients with early-stage COPD: a cross-sectional study. *npj Primary Care Respiratory Medicine*.

[12] Meijer K., Annegarn J., Lima Passos V. (2014). Characteristics of daily arm activities in patients with COPD. *European Respiratory Journal*.

[13] Baarends E. M., Schols A. M., Slebos D. J., Mostert R., Janssen P. P., Wouters E. F. (1995). Metabolic and ventilatory response pattern to arm elevation in patients with COPD and healthy age-matched subjects. *European Respiratory Journal*.

[14] Annegarn J., Meijer K., Passos V. L. (2012). Problematic activities of daily life are weakly associated with clinical characteristics in COPD. *Journal of the American Medical Directors Association*.

[15] Bendixen H. J., Ejlersen Wæhrens E., Wilcke J. T., Sørensen L. V. (2014). Self-reported quality of ADL task performance among patients with COPD exacerbations. *Scandinavian Journal of Occupational Therapy*.

[16] Nakken N., Janssen D. J. A., van den Bogaart E. H. A. (2017). Patient versus proxy-reported problematic activities of daily life in patients with COPD. *Respirology*.

[17] Criswell E. (2010). *Cram’s Introduction to Surface Electromyography*.

[18] Dolmage T. E., Maestro L., Avendano M. A., Goldstein R. S. (1993). The ventilatory response to arm elevation of patients with chronic obstructive pulmonary disease. *Chest*.

[19] Couser J. I., Martinez F. J., Celli B. R. (1992). Respiratory response and ventilatory muscle recruitment during arm elevation in normal subjects. *Chest*.

